# Association of Macrolide Resistance Genotypes and Synergistic Antibiotic Combinations for Combating Macrolide-Resistant MRSA Recovered from Hospitalized Patients

**DOI:** 10.3390/biology10070624

**Published:** 2021-07-06

**Authors:** Amr S. Bishr, Salma M. Abdelaziz, Ibrahim S. Yahia, Mahmoud A. Yassien, Nadia A. Hassouna, Khaled M. Aboshanab

**Affiliations:** 1 Department of Microbiology and Immunology, Faculty of Pharmacy, Ain Shams University, Organization of African Unity St., Cairo 11566, Egypt; amr.shaker@pharma.asu.edu.eg (A.S.B.); salma_mustafa87@pharm.asu.edu.eg (S.M.A.); mahmoud.yassien@pharma.asu.edu.eg (M.A.Y.); nadia.hassouna@pharma.asu.edu.eg (N.A.H.); 2 Research Center for Advanced Materials Science (RCAMS), King Khalid University, Abha P.O. Box 9004, Saudi Arabia; ihussein@kku.edu.sa; 3 Advanced Functional Materials & Optoelectronic Laboratory (AFMOL), Department of Physics, Faculty of Science, King Khalid University, Abha P.O. Box 9004, Saudi Arabia; 4 Nanoscience Laboratory for Environmental and Bio-Medical Applications (NLEBA), Semiconductor Lab., Physics Department, Faculty of Education, Ain Shams University, Roxy, Cairo 11757, Egypt

**Keywords:** macrolide resistance, methicillin-resistant *Staphylococcus aureus*, coagulase-negative staphylococci, cMLS, iMLS, MS, *erm*A, *erm*C, *msr*A

## Abstract

**Simple Summary:**

Macrolide-resistant methicillin-resistant *Staphylococcus aureus* (MAC-MRSA) is one of the most clinically relevant pathogens due to its significant ability of resistance acquisition to different antimicrobial agents and narrow therapeutic options. This study aimed to evaluate antimicrobial susceptibility and the use of different combinations of azithromycin with other antibiotics as well as studying the correlation of MAC resistance genotypes and antimicrobial agents that provided synergy when they were combined with azithromycin. Azithromycin (AZM) combinations with either linezolid, ceftriaxone, gentamicin, or cefotaxime provided synergy in 42.1%, 44.7%, 31.6% and 7.9% of the 38 MAC-MRSA isolates, respectively. Statistical analysis showed significant association between the presence of the *erm*A genotype and the synergism of AZM + ceftriaxone and AZM + gentamicin; the presence of the *erm*C genotype and the synergism between AZM and gentamicin; the presence of the *msr*A genotype and the synergism between AZM and ceftriaxone; and the presence of the *erm*A/*msr*A genotype and the synergism between AZM and cefotaxime. The obtained findings will guide clinicians in better choosing the antibiotic combinations required for combating MAC-MRSA clinical isolates. However, the promising synergistic antibiotic combinations must be re-evaluated in vivo using an appropriate animal model.

**Abstract:**

Macrolide-resistant methicillin-resistant *Staphylococcus aureus* (MAC-MRSA) is one of the most clinically relevant pathogens due to its significant ability of resistance acquisition to different antimicrobial agents. This study aimed to evaluate antimicrobial susceptibility and the use of different combinations of azithromycin with other antibiotics for combating MAC resistance. Seventy-two *Staphylococci* (38.5%) (n = 187), showed resistance to MACs; of these, 53 isolates (73.6%, n = 72) were *S. aureus* and 19 (26.4%, n = 72) were coagulase-negative staphylococci (CoNS). Out of the 53 *S. aureus* and 19 CoNS isolates, 38 (71.7%, n = 53) and 9 (47.4%, n = 19) were MRSA and methicillin-resistant CoNS, respectively. The constitutive MACs, lincosamides and streptogramin-B (cMLS) comprised the predominant phenotype among *S. aureus* isolates (54.7%) and CoNS isolates (78.9%). The PCR analysis showed that the *erm*C gene was the most prevalent (79.2%), followed by *msr*A (48.6%), and *erm*A (31.9%). Azithromycin combinations with either linezolid, ceftriaxone, gentamicin, or cefotaxime provided synergy in 42.1%, 44.7%, 31.6% and 7.9% of the 38 MAC-MRSA isolates, respectively. Statistical analysis showed significant association between certain MAC resistance genotypes and the synergistic effect of certain azithromycin combinations (*p* value < 0.05). In conclusion, azithromycin combinations with either linezolid, or ceftriaxone showed synergism in most of the MAC-resistant MRSA clinical isolates.

## 1. Introduction

Antimicrobial resistance is deemed to be a serious concern to modern medicine, as it certainly reduces the possibility of successful treatment of infectious diseases [1]. Macrolide-resistant-methicillin-resistant *Staphylococcus aureus* (MAC-MRSA) is considered to be one of the most common causes of healthcare-associated infections, due to higher morbidity and mortality, prolongation of hospitalization period and increase in health care costs, in comparison with infections caused by methicillin-susceptible strains [2]. There is a wide range of MRSA infections, ranging from mild skin and soft tissue infections to life-threatening diseases, such as endocarditis, osteomyelitis, and pneumonia [3,4]. Owing to their prominent ability of resistance acquisition to different antimicrobial agents, in addition to the difficulty of treatment of this pathogen, MRSA strains create a major threat to public health [5,6]. Due to the limited use of beta-lactams for treatment of MRSA infections, non-beta-lactam antimicrobials, such as aminoglycosides, fluoroquinolones, glycopeptides, lincosamides, lipopeptide and MACs, are required for the treatment of staphylococcal infections. Nevertheless, this can be contradicted by the development of resistance mechanisms by MRSA isolates to overcome the high concentrations of these antimicrobials [5,7].

MAC antibiotics belong to one of the most common classes of clinically important antibiotics used in the management of infections produced by Gram-positive bacteria [8]. For many years, MACs have been the major alternative to β-lactams for the treatment of infections due to Gram-positive pathogens. However, the worldwide development of MAC resistance has sometimes constrained the use of these antibiotics to certain indications [9]. Resistance of MRSA strains to MACs is correlated with various genotypic and phenotypic mechanisms, including alterations in ribosomal binding site (*ermA*, *ermB* and *ermC*), confering resistance to MACs, lincosamides and type B streptogramins [7]. In addition, active efflux pumps, such as those encoded by the *msrA* and *msrB genes* [1], which impart resistance to MACs and type B streptogramins, are also involved in MAC resistance. These resistance processes reduce the medicinal treatment opportunities available for combating MRSA infections. Therefore, this study aimed to evaluate antimicrobial susceptibility and identify the MAC-resistance coding genes in MRSA clinical isolates obtained from hospitalized patients in one of the major tertiary care hospitals in Egypt, and to study the use of different azithromycin combinations with other antibiotics for the purpose of combating MAC-MRSA associated infection.

## 2. Materials and Methods

### 2.1. Clinical Isolates

A total of 187 *Staphylococcus* clinical isolates were obtained from different clinical specimens collected from hospitalized patients. These specimens included pus (87; 46.5%), blood (32; 17.1%), sputum (54; 28.9%) and bronchoalveolar lavage (BAL) (14; 7.5%) discharged from the Microbiology diagnostic laboratories of Al-Demerdash Hospital, Cairo, Egypt. *S. aureus* isolates were distinguished from other staphylococci by giving yellow colonies after culture on mannitol salt agar and giving positive coagulase test results [10,11]. *S. aureus* ATCC^®^ 2592 (VA, USA) standard strain was used for the quality control of antimicrobial susceptibility tests. MRSA isolates were detected using cefoxitin discs (30 μg) obtained from Bioanalyse, Turkey, and confirmed by detection of the *mec*A gene via PCR [12,13]. The study was approved by the Faculty of Pharmacy, Ain Shams University Research Ethics Committee (ENREC-ASU-Nr. 94b), in which both informed and written consents were obtained from patients or patients’ parents after elucidating the study purpose.

### 2.2. Antimicrobial Susceptibility Testing

The collected clinical isolates were screened for their susceptibility to four antibiotics: erythromycin (15 μg), clindamycin (2 μg), azithromycin (15 μg) and spiramycin (10 μg) via the Kirby–Bauer disc diffusion method [14], and the isolates were classified as susceptible, of intermediate susceptibility or resistant according to standard zone diameter break points stated in the CLSI guidelines 2018. The antibiotic discs were purchased from Oxoid^®^, Hampshire, United Kingdom The inducible resistance phenotype was detected by double-disc diffusion test [15,16].

### 2.3. Determination of Minimum Inhibitory Concentration

The MAC-resistance of the resistant isolates was further confirmed by determination of the MIC of erythromycin (ERY) and azithromycin (AZM) against the resistant isolates via the broth microdilution method using 96-well microtiter plates [17]. The antibiotic powders (ERY and AZM) were purchased from Sigma-Aldrich. (MA, USA).

### 2.4. Molecular Detection of MAC Resistance Genes

#### 2.4.1. Chromosomal DNA Extraction of the Tested Isolates

The chromosomal DNA of the resistant isolates was extracted using the Zyppy™ (Irvine, MA, USA) Genomic DNA purification Kit according to the manufacturer’s recommendations and was used as a template for PCR.

#### 2.4.2. PCR Amplification of MAC Resistance Genes

The PCR was performed in the thermal cycler (Nyx Technik, MA, USA). The reaction mixture of volume (25 µL) was prepared according to the protocol provided by the Fermentas Master Mix kit (Table 1). The MAC-resistant isolates were screened for presence of erythromycin ribosomal methylase genes (*erm*A, *erm*C) and the MAC-streptogramin resistance gene (*msr*A) using the primers listed in Table 2.

#### 2.4.3. Agarose Gel Electrophoresis

The PCR products were visualized through 0.8% agarose gel electrophoresis containing 0.5 μg/mL ethidium bromide using 1 kb DNA ladder (Thermoscientific, Waltham, MA, USA) [15]. Purification of the PCR products was carried out using the GeneJET™ PCR Purification kit (Fermentas, Waltham, MA, USA) at Sigma Scientific Services Company, Egypt.

### 2.5. Phenotypic Analysis of the Recovered MRSA Isolates Using Heatmap Signature Analysis

The MAC resistance profiles, MAC resistance genes and detected MAC phenotypes among MRSA isolates were used to generate a dendrogram showing the heatmap signature of the isolates. It was generated by Morpheus online software (https://software.broadinstitute.org/morpheus/, accessed on 14 April 2021) using Euclidean distances.

### 2.6. Checkerboard Titration Method for Studying the Effect of Combinations between MACs and Different Antimicrobial Agents

A screening test for potential synergism between MACs and other antibiotics against MAC-resistant MRSA isolates was carried out via a broth microdilution checkerboard assay using 96-well U-bottom microtiter plates in an eight-by-eight-well configuration [21]. Each antibiotic was two-fold serial diluted with the concentration range from 0.063× MIC to 4× MIC. The inoculum of the tested isolates was prepared by emulsifying overnight colonies from an agar medium into saline. A volume of bacterial suspension equal to the volume of diluted antibiotic solution was added to each well of the microtiter plate to achieve the final microbial concentration in each well of 5×10^5^ cfu/mL. Positive growth controls which did not contain any antibiotics and negative growth controls were included in each assay.

The combination of AZM with linezolid (LZD), cefotaxime (CTX), ceftriaxone (CEF), amikacin (AMK) and gentamycin (GEN). Microtiter plates were incubated for 18–24 h at 37 °C. The results of checkerboard assay were interpreted by calculation of the total fractional inhibitory concentration index (ΣFIC index) as previously determined [21,22].

### 2.7. Statistical Analysis

Statistical analysis of the data was carried out using IBM SPSS Statistics software for Windows v.20.0 (IBM Corp., Armonk, NY, USA). Qualitative data were expressed as frequency and percentage. Chi-square test was used for comparison of categorical variables. All tests were two-tailed, and *p-*value < 0.05 was considered as statistically significant.

## 3. Results

### 3.1. The Antimicrobial Susceptibility Testing of the Collected Staphylococci (n = 72)

**Figure 1:**
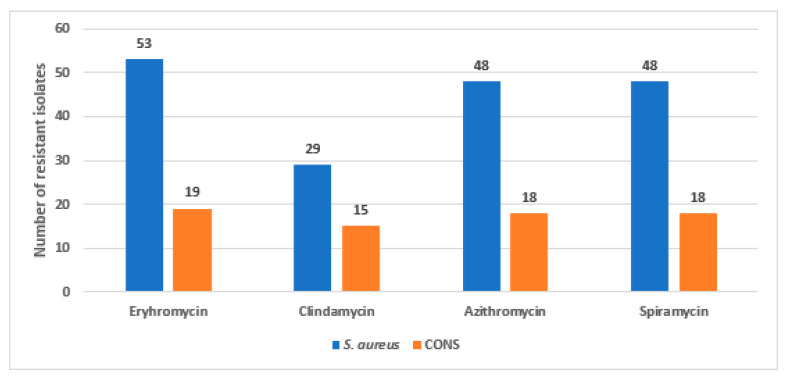
The sensitivity of the resistant *Staphylococcus* isolates (n = 72) against the tested antibiotics.

The antimicrobial susceptibility test by the disc diffusion method showed that 72 *Staphylococcus* isolates, out of the collected isolates, displayed MAC resistance, where 53 isolates were identified as *S. aureus*, while the other 19 isolates were CoNS. The sensitivity of the resistant *Staphylococcus* sp. isolates against the tested MAC antibiotics are shown in Figure 1. Among the resistant *S. aureus* isolates, thirty-eight MRSA isolates were detected, which were recovered from 20 pus, 10 blood, 6 sputum and 2 bronchoalveolar lavage (BAL) clinical specimens.

The minimum inhibitory concentration (MIC) of both erythromycin (ERY) and azithromycin (AZM) were determined for MAC-resistant *S. aureus* isolates. It was found that 92.5% (n = 49) and 94.3% (n = 50) of the 53 *S. aureus* isolates showed high levels of resistance. For the MAC resistant *S. aureus* (n = 53), the MIC_50_ was 2000 and 1024 µg/mL, and the MIC_90_ was 3500 and 4096 µg/mL for ERY (MIC range, 250–4000 µg/mL) and AZM (MIC range, 512–8192 µg/mL), respectively. For the MAC resistant CoNS isolates (n = 19), the MIC_50_ was 2000 and 2048 µg/mL, and the MIC_90_ was 4000 and 4096 µg/mL for ERY (MIC range, 250–8000 µg/mL) and AZM (MIC range, 512–8192 µg/mL), respectively.

### 3.2. MAC-Resistance Phenotypes

**Figure 2:**
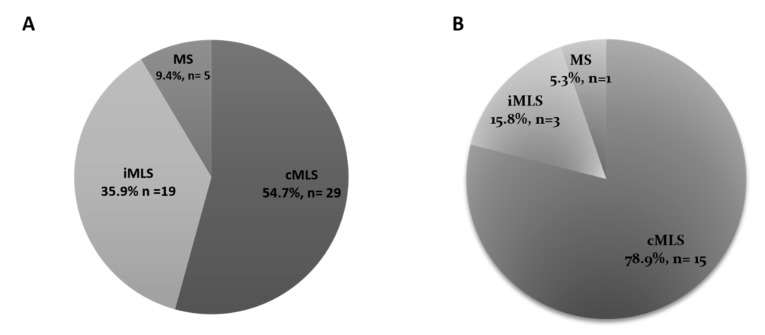
Distribution of MAC-resistance phenotypes among the resistant *S. aureus* isolates (**A**), and coagulase-negative *Staphylococcus* sp. isolates (**B**). The cMLS phenotype was the most frequent with 54.7% and 78.9% prevalence among *S. aureus* and CoNS isolates, respectively.

The distribution of MAC resistance phenotypes among the *S. aureus* and CoNS resistant isolates is depicted in Figure 2. The inducible resistance phenotype (iMLS) was determined by showing the D-shaped inhibition zone around clindamycin discs.

MAC-resistant *Staphylococcus* sp. isolates were tested for their susceptibility to methicillin, where 38 MRSA isolates were detected among *S. aureus* isolates, and 9 MR-CoNS isolates were detected among CoNS isolates. Regarding the distribution of MAC-resistance phenotypes among MRSA isolates, it was found that the constitutive resistance phenotype was the predominant phenotype, followed by the inducible, then the MS phenotype. In addition, all methicillin-resistant CoNS isolates were shown to exhibit the cMLS phenotype. The statistical analysis showed that there is a significant correlation between the methicillin-resistance and the type of MAC-resistance phenotype among *S. aureus* isolates with a *p* value of 0.009 (<0.05) (Table 3).

### 3.3. Molecular Detection of MAC Resistance Genes among MAC Resistant Staphylococci

**Figure 3:**
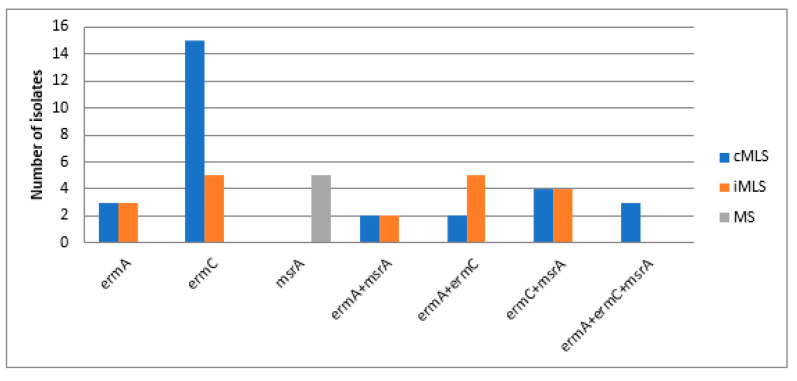
Distribution of MAC-resistance genotypes among the resistant *S. aureus* isolates. *erm*A: erythromycin 23S ribosomal methylase gene A. *erm*C: erythromycin 23S ribosomal methylase gene C, *msr*: macrolide-streptogramin resistance gene.

**Figure 4:**
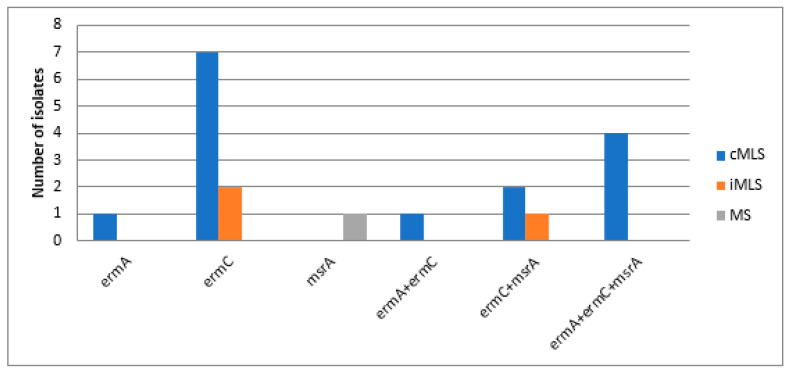
Distribution of MAC-resistance genotypes among the resistant coagulase-negative *Staphylococcus* sp. isolates. *erm*A: erythromycin 23S ribosomal methylase gene A. *erm*C: erythromycin 23S ribosomal methylase gene C, *msr*: macrolide-streptogramin resistance gene.

Chromosomal DNA, extracted from the resistant isolates, were used as templates for PCR detection of the presence of the MAC-resistance coding genes (23S rRNA methylase A (*erm*A), 23S rRNA methylase C (*erm*C) and MAC-streptogramin resistance gene (*msr*A), using the primers listed in Table 4. The PCR results showed that *erm*C was the most frequently occurring gene (79.2%), followed by the *msr*A gene (48.6%), then the *erm*A *gene* (31.9%). Distribution of MAC-resistance coding genes among resistant *S. aureus* and CoNS isolates is demonstrated in Figure 3 and Figure 4. In addition, different resistance phenotypes, and the distribution of different MAC resistance genes among MRSA isolates, are shown in Table S1 (Supplementary Materials).

### 3.4. Phenotypic Analysis of the Recovered MRSA Isolates Using Heatmap Signature Analysis

**Figure 5:**
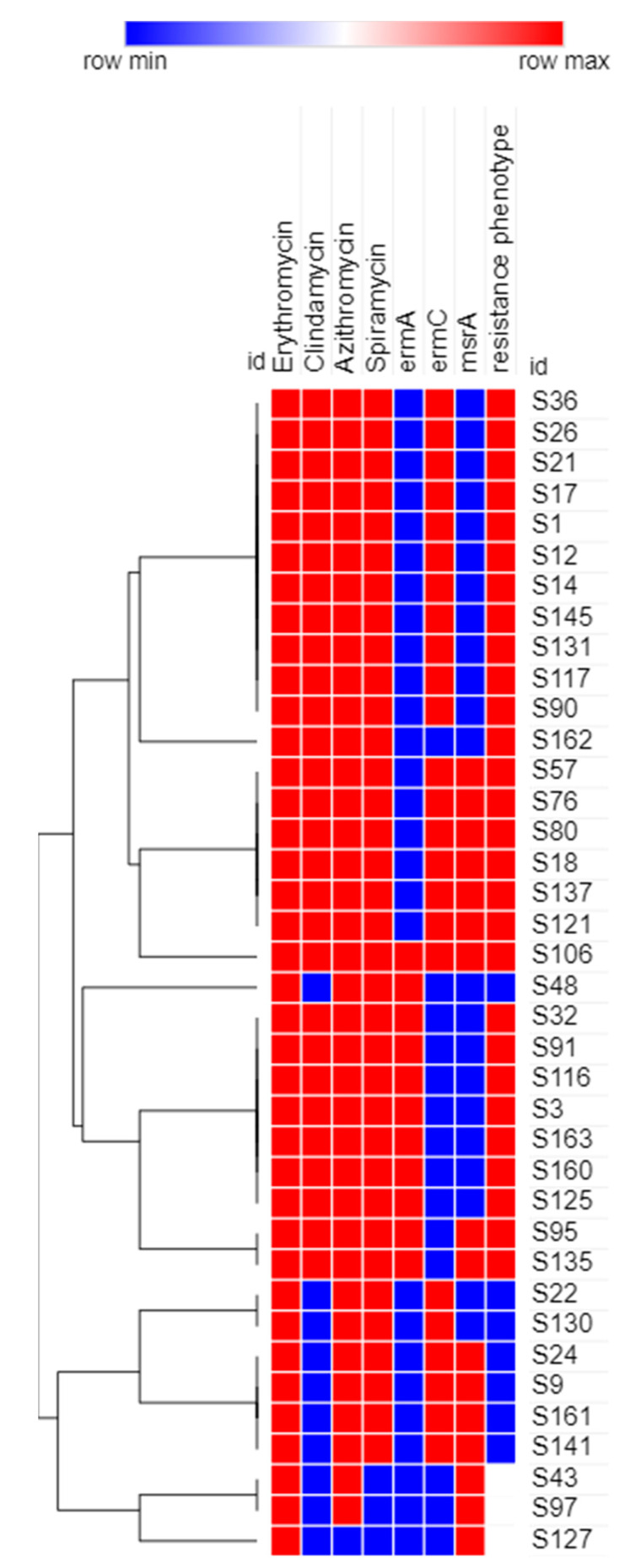
Dendrogram heatmap of MRSA isolates (n = 38) in the study based on their antimicrobial resistance patterns, and resistance genotypes and phenotypes. This heatmap was generated using Morpheus online software with Euclidean distances (https://software.broadinstitute.org/morpheus/, accessed on 14 April 2021).

The results obtained proved the diversity of isolates with respect to their phenotypic characteristics (Figure 5). The recovered 38 MRSA isolates were clustered into 11 clusters based on the tested phenotypic characteristics.

### 3.5. Effect Azithromycin Combinations with Different Antimicrobial Agents on MAC MRSA Isolates (n = 38)

The obtained findings revealed that the combinations of AZM + linezolid (LZD), AZM + ceftriaxone (CEF), AZM + gentamicin (GEN) and AZM + cefotaxime (CTX) showed synergistic effects in 42.1%, 44.7%, 31.6% and 7.9% of the 38 MRSA isolates, respectively (FIC < 0.5). The Effects of azithromycin combinations with different antimicrobial agents against MRSA isolates by the checkerboard method are illustrated in Table S2 (Supplementary Materials).

### 3.6. Statistical Analysis

As shown in Table 4, statistical analysis has shown a statistically significant association between certain MAC resistance genotypes and the synergistic effect between different AZM combinations (*p* value < 0.05). Calculation of the Pearson Chi-square value showed significant association between the presence of the *erm*A genotype and the synergism between AZM and CEF, and between AZM and GEN; and the presence of the *erm*C genotype and the synergism between AZM and GEN. There was also significant association between the presence of the *msr*A genotype and the synergism between AZM and CEF; and the presence of the *erm*A/*msr*A genotype and the synergism between AZM and CTX.

## 4. Discussion

Antimicrobial resistance can lead to the emergence and dissemination of multidrug resistant microorganisms, resulting in a serious threat to patient health [23]. The multidrug resistant infections are widespread all over the world and are considered as a crisis that could have “catastrophic consequences” [23,24,25]. In this study, a total number of 187 *Staphylococcus* clinical isolates were collected from different clinical specimens discharged from the Microbiology laboratory of Al-Demerdash Hospital, Cairo, Egypt.

There are three MAC resistance phenotypes, which are: the cMLS, iMLS and MS phenotypes. It was found that cMLS was the predominant phenotype among the resistant isolates in both *S. aureus* and CoNS isolates. The distribution of the MAC-resistance phenotypes in our study agreed with the study carried out in China by Yao and co-workers, where the cMLS phenotype was the most predominant (95, n = 96) over the iMLS phenotype (1, n = 96) among *S. aureus* isolates from different clinical specimens [26]. The same distribution of resistance phenotypes also was observed in the study carried out in Abakaliki, Nigeria, where cMLS was found in 20.5% (8, n = 40) of the tested *S. aureus* isolates, while the iMLS phenotype was present in 15.4% (6, n = 40) of the tested isolates [27]. The results of this study, regarding the ranking of the resistance phenotypes, agreed with the study conducted in India, where the cMLS phenotype was found in 47.22% (51, n = 108) of tested CoNS isolates isolated from different clinical specimens, followed by nearly equal percentages of both the iMLS and MS phenotypes (26.85% and 25.92%, respectively) [28].

The prevalence of MRSA has significantly elevated all over the world in the last two decades. Its proportion varies substantially depending on the geographical region and the human population [29]. The highest percentage of methicillin-resistant (MR) isolates was found in pus specimens, followed by blood, then sputum and BAL specimens. The predominance of the percentage of MRSA from pus specimens in our study agreed with the findings from studies conducted by Kaur and co-workers in 2015, and Majhi and co-workers in 2016, where MRSA was most frequently isolated from the pus specimen (13.56% and 53.1%, respectively) [30,31]. The distribution of MAC-resistance phenotypes among MRSA isolates in our study agreed with the findings from another study carried out in Nepal in 2017, where the cMLS phenotype was the most frequent phenotype (79/270, 29.25%), followed by the iMLS phenotype with a prevalence of (31/270, 11.48%). Although the prevalence of the iMLS phenotype among the isolates in our study was deemed to be low, this study exhibited higher percentages of resistance to both erythromycin and clindamycin among MRSA as compared to other studies [32,33]. The results of our study are strongly supported by the findings of another study conducted in Iran in 2019, where the cMLS phenotype was the most occurring MAC-resistance phenotype among the MAC-resistant MRSA isolates, with a prevalence of 56.2% (27, n = 48) among the tested isolates, followed by the iMLS phenotype, which was found in 22.9% (11, n = 48) of the tested isolates, and then the MS phenotype that was present in only 16.6% (8, n = 48) of the MRSA isolates [7]. It was found that *erm*C was the predominant gene among the tested isolates (79.2%, 57/72), followed by the *msr*A gene (48.6%, 35/72), then by the *erm*A gene (31.9%, 23/72). The results of our study are strongly supported by the findings of another study conducted in Iran in 2019 [7]. In the latter study, the *erm*C gene was the most frequently occurring one among the tested S. aureus isolates, regardless of whether the MAC-resistance phenotype was constitutive or inducible. Among the isolates exhibiting the cMLS phenotype, it was found that the *erm*C gene was the most frequently occurring gene (44.4%, 12 isolates), followed by *erm*A (25.9%, 7 isolates), then *erm*B (18.5%, 5 isolates). In isolates exhibiting the iMLS phenotype, it was found that ermC showed the greatest predominance, with a prevalence of 81.8% (9 isolates), followed by *erm*B with 63.6% (7 isolates), then *erm*A with 54.5% prevalence (6 isolates) [7]. The high prevalence and the predominance of the *erm*C gene over the other genes accountable for MAC-resistance can be attributed to the findings of Fluit et al. who reported the presence of the *erm*C gene on small plasmid, resulting in the ease of its transmission and greater dissemination from resistant to susceptible strains compared to other genes [6,33].

Another important aim of our study was to investigate the correlation between the genotypes and phenotypes of the recovered resistant *Staphylococcus* sp. isolates collected from hospitalized patients with serious infections at Al-Demerdash hospital in Egypt. Our findings revealed that all isolates showing resistance to both MACs and lincosamides; MLS phenotypes, either constitutive or inducible; were found to harbor at least one type of *erm* gene (*erm*A or *erm*C). Different factors, such as geographical region and variations in population, can determine the resistance phenotype; specifically, whether it is constitutive or inducible [34]. The difference between both phenotypes is in the type of methylase mRNA produced, where active mRNA is produced in the absence of an inducer in the case of the cMLS phenotype, and inactive mRNA is produced in bacteria showing the iMLS phenotype, which becomes active only in the presence of the MAC inducer [35]. The genotype of the six isolates exhibiting the MS resistance phenotype showed that only *msr*A was present, which explains the resistance of these isolates only to MACs, but not to clindamycin. To study the clonal relationship of the collected MAC MRSA isolates, a heatmap dendrogram was created based on the obtained phenotypic and genotypic characteristics of the respective isolates. The results obtained proved the diversity of isolates with respect to their phenotypic characteristics. Our findings revealed that the MAC-MRSA isolates (n = 38) were clustered into 11 clusters based on the tested phenotypic characteristics. The obtained results provide more insight into the phenotypic relatedness of the isolates and proved the possibility that some isolates may be nosocomially transmitted.

Consequently, the increase in virulent and resistant strains of *S. aureus* encourages the scientific community to develop new therapeutic strategies [36,37] to be used in combination with the therapies currently in use, in order to enhance the antibacterial activity and counteract the evolution of resistance. Therefore, the effect of different combinations of AZM with different antimicrobial agents including LZD, CEF, CTX, AMI and GEN on MAC-resistant MRSA isolates was studied using the checkerboard assay. The results revealed that AZM + LZD, AZM + CEF, AZM + GEN and AZM + CTX showed synergistic effect in (42.1%, n = 16), (44.7%, n = 17), (31.6%, n = 12) and (7.9%, n = 3) of the 38 MRSA isolates, respectively (FIC < 0.5), while additive effect was detected when AZM was used in combination with AMI on the same isolates (0.5 < ΣFICI < 1). Statistical analysis has shown a statistically significant association between some MAC resistance genotypes and the synergistic effect between different AZM combinations (*p* value < 0.05). Calculation of the Pearson Chi-square value showed significant association between the presence of the *erm*A genotype and the synergism between AZM and CEF, and between AZM and GEN; and the presence of the *erm*C genotype and the synergism between AZM and GEN. There was also significant association between the presence of the *msr*A genotype and the synergism between AZM and CEF; and the presence of the *erm*A and *msr*A genotypes and the synergism between AZM and CTX. This result agreed with the results of other studies, such as one conducted by Singh and co-workers in 2018, where a synergistic effect was detected between AZM + CEF and AZM + GEN when used against *N*. *gonorrhoeae* isolates [38]. The synergistic effects between AZM and either CEF or GEN can be explained by the difference in either the mechanism or target site of action, respectively. AZM and GEN act by inhibition of the 50S and 30S bacterial ribosomal subunits, while CEF acts by damaging of the bacterial cell wall, thus increasing the uptake of AZM, resulting in the production of a more potent effect than if each antibiotic was used alone [38]. Our results regarding antibiotic susceptibility and MIC revealed high levels of resistance of MRSA isolates to AZM when it was used alone as a monotherapy. However, different combinations of AZM and other antibiotics showed a significant synergistic effect against MRSA, as demonstrated by checkerboard assay, particularly for AZM + LZD. The MIC of LZD decreased significantly when it was used in combination with AZM, as shown in the results. This will, of course, be of significant medical importance as decreased dosages of LZD will decrease its potential side effects in humans. Accordingly, more studies, including preclinical and clinical studies, are mandatory to validate the in vivo synergistic effect of AZM on anti-MRSA drugs and evaluate its use in combined therapy.

## 5. Conclusions

This study revealed high percentages of resistance of the collected *Staphylococcus* sp. isolates, especially MRSA strains, to MAC antibiotics. Statistical analysis showed significant association between some MAC resistance genotypes and the synergistic effect of different AZM combinations. The MIC of LZD decreased significantly when it was used in combination with AZM. The findings of this study will be of value in guiding clinicians to better choose the antibiotic combinations required for combating MAC-MRSA clinical isolates.

## Figures and Tables

**Table 1:** Conditions of the PCR. (X: annealing temperature was adjusted according to the melting temperature of the used primers).

Step	Temperature °C	Time	No. of cycles
Initial denaturation	95	5 min	1
Denaturation	95	30 s	30
Annealing	X	30 s	
Extension	72	1 min	
Final extension	72	5 min	1

**Table 2:** The primer sequences used in this study and the expected PCR product sizes.

Primer	Target Gene	Primer Sequence (5’⟶3’)	Ta (°C)	PCR Product (Kb)	Reference
ErmA-f	*erm*A	TATCTTATCGTTGAGAAGGGATT	50	139	[18]
ErmA-r		CTACACTTGGCTTAGGATGAAA			
ErmC-f	*erm*C	AATCGTCAATTCCTGCATGT	51	299	[19]
ErmC-r		TAATCGTGGAATACGGGTTTG			
Msr-f	*msr*A	TCCAATCATAGCACAAAATC	47	163	[18]
Msr-r		AATTCCCTCTATTTGGTGGT			
MecA-f	*mec*A	AAAATCGATGGTAAAGGTTGGC	50	533	[20]
MecA-r		AGTTCTGGAGTACCGGATTTGC			

*erm*A: erythromycin 23S ribosomal methylase gene A. *erm*C: erythromycin 23S ribosomal methylase gene C. *msr* A: MAC-streptogramin resistance gene. *mec*A encodes the protein PBP2A (penicillin-binding protein 2A), which confirms the presence of MRSA isolates.

**Table 3:** Correlation between the methicillin-resistance and the type of MAC-resistance phenotype *S. aureus* isolates.

Isolates	Resistance Phenotype, n (%)			*p* Value
	cMLS	iMLS	MS	
MRSA (n = 38)	28 (73.7%)	7 (18.4%)	3 (7.9%)	0.009 (Significant)
MSSA (n = 15)	4 (26.7%)	10 (66.6%)	1 (6.7%)	

**Table 4:** Statistical association between MAC resistance genotypes of MRSA isolates and the synergistic effect of different azithromycin combinations and their respective *p* values.

Resistance Genotype	No. of Isolates	*Pearson Chi square* (*p*)				
		AZM + LIN	AZM + CEF	AZM + GEN	AZM + AMI	AZM + CTX
*erm*A	8	0.189	0.050	0.034	NA	0.351
*erm*C	14	0.452	0.393	0.08	NA	0.168
*msr*A	3	0.369	0.045	0.173	NA	0.597
*erm*A, *msr*A	2	0.215	0.191	0.324	NA	0.023
*erm*C, *msr*A	10	0.099	0.275	0.9	NA	0.098
*erm*A, *erm*C, *msr*A	1	0.387	0.362	0.491	NA	0.767

AZM: Azithromycin, LZD: Linezolid, GEN: Gentamicin, AMI: Amikacin, CEF: Ceftriaxone, and CTX: Cefotaxime. NA (not available because of the absence of synergism of this combination), *erm*A: erythromycin 23S ribosomal methylase gene A. *erm*C: erythromycin 23S ribosomal methylase gene C, *msr*: macrolide-streptogramin resistance gene.

## Data Availability

Data are available within the article.

## Supplementary Materials

The following are available online at https://www.mdpi.com/article/10.3390/biology10070624/s1, Table S1: Different resistance genotypes and phenotypes among MRSA isolates; Table S2: Effects of azithromycin combinations with different antimicrobial agents against MRSA isolates using the checkerboard assay.
