# Supplementary material for: Association of Macrolide Resistance Genotypes and Synergistic Antibiotic Combinations for Combating Macrolide-Resistant MRSA Recovered from Hospitalized Patients
Source: Biology (Basel). 2021 Jul 6;10(7):624. doi: 10.3390/biology10070624 (PMC8301042; doi:10.3390/biology10070624)
Supplement: Supplementary file 1 [file biology-10-00624-s001.zip › biology-1263493-supplementary.pdf]

Table S1: Different resistance genotypes and phenotypes among MRSA isolates

Isolate number	*erm*A	*erm*C	*msr*A	Resistance phenotype
S1	-	+	-	cMLS
S3	+	-	-	cMLS
S9	-	+	+	iMLS
S12	-	+	-	cMLS
S14	-	+	-	cMLS
S17	-	+	-	cMLS
S18	-	+	+	cMLS
S21	-	+	-	cMLS
S22	-	+	-	iMLS
S24	-	+	+	iMLS
S26	-	+	-	cMLS
S32	+	-	-	cMLS
S36	-	+	-	cMLS
S43	-	-	+	MS
S48	+	-	-	iMLS
S57	-	+	+	cMLS
S76	-	+	+	cMLS
S80	-	+	+	cMLS
S90	-	+	-	cMLS
S91	+	-	-	cMLS
S95	+	-	+	cMLS
S97	-	-	+	MS
S106	+	+	+	cMLS
S116	+	-	-	cMLS
S117	-	+	-	cMLS
S121	-	+	+	cMLS
S125	+	-	-	cMLS
S127	-	-	+	MS
S130	-	+	-	iMLS
S131	-	+	-	cMLS
S135	+	-	+	cMLS
S137	-	+	+	cMLS
S141	-	+	+	iMLS
S145	-	+	-	cMLS
S160	+	-	-	cMLS
S161	-	+	+	iMLS
S162	-	+	-	cMLS
S163	+	-	-	cMLS

cMLS: constitutive macrolide, lincosamide and streptogramin; iMLS: inducible macrolide, lincosamide and streptogramin; MS: macrolide and streptogramin; +: present; -: absent

Table S2: Effects of azithromycin combinations with different antimicrobial agents against MRSA isolates (n =38) using the checkerboard assay. (All values in µg/ml).

	AZM+LIN	∑FIC	I	AZM+CEF	∑FIC	I	AZM+GEN	∑FIC	I	AZM+AMI	∑FIC	I	AZM+CTX	∑FIC	I
**S1**	256/0.25	0.5	S	256/64	0.37	S	128/8	0.625	A	128/8	0.625	A	256/1024	0.75	A
**S3**	128/0.5	0.37	S	256/128	0.37	S	256/4	0.5	S	256/8	0.75	A	512/256	0.625	A
**S9**	256/1	0.625	A	1024/64	0.56	A	256/4	0.37	S	256/8	0.625	A	128/1024	0.56	A
**S12**	256/0.5	0.37	S	512/256	0.5	S	256/8	0.625	A	256/8	0.625	A	1024/512	0.75	A
**S14**	128/1	0.56	A	256/512	0.625	A	1024/4	0.75	A	256/8	0.625	A	1024/256	0.625	A
**S17**	256/0.25	0.5	S	256/64	0.37	S	256/4	0.5	S	64/8	0.56	A	512/256	0.625	A
**S18**	128/0.5	0.625	A	128/128	0.37	S	128/8	0.625	A	512/1	0.56	A	512/128	0.56	A
**S21**	128/0.5	0.625	A	128/256	0.625	A	512/1	0.56	A	64/8	0.56	A	256/1024	0.75	A
**S22**	256/0.25	0.5	S	512/256	1	A	128/8	0.625	A	256/8	0.75	A	512/1024	1	A
**S24**	512/0.5	0.75	A	512/64	0.56	A	128/16	0.625	A	256/16	0.75	A	512/512	0.625	A
**S26**	256/0.25	0.5	S	256/64	0.37	S	128/8	0.625	A	128/8	0.625	A	256/1024	0.75	A
**S32**	256/1	0.625	A	1024/64	0.56	A	256/4	0.37	S	256/8	0.625	A	128/1024	0.56	A
**S36**	128/0.5	0.625	A	128/256	0.625	A	512/1	0.56	A	64/8	0.56	A	256/1024	0.75	A
**S43**	256/0.5	0.37	S	512/256	0.5	S	256/8	0.625	A	256/8	0.625	A	1024/512	0.75	A
**S48**	256/0.5	0.5	S	128/256	0.37	S	256/4	0.75	A	512/8	1	A	512/128	0.625	A
**S57**	128/0.5	0.625	A	256/128	0.5	S	128/4	0.37	S	512/8	1	A	256/512	0.75	A
**S76**	128/1	0.56	A	1024/512	1	A	1024/2	0.75	A	512/4	0.75	A	512/512	0.5	S
**S80**	256/1	0.625	A	256/512	0.625	A	512/4	0.5	S	1024/2	0.75	A	1024/512	1	A
**S90**	512/0.5	0.5	S	512/512	0.75	A	1024/4	1	A	1024/2	0.75	A	512/512	0.75	A
**S91**	256/0.5	0.5	S	256/128	0.37	S	128/8	0.625	A	512/16	1	A	512/512	0.625	A
**S95**	128/1	0.56	A	1024/512	1	A	1024/1	0.56	A	512/4	0.75	A	512/256	0.37	S
**S97**	64/1	0.625	A	128/256	0.5	S	64/4	0.37	S	128/4	0.75	A	256/128	0.625	A
**S106**	512/0.5	0.625	A	512/256	0.625	A	2048/1	0.56	A	256/8	0.56	A	1024/1024	0.75	A
**S116**	32/0.5	0.31	S	64/256	0.37	S	64/4	0.25	S	256/128	0.53	A	256/1	0.625	A
**S117**	128/0.5	0.625	A	128/256	0.625	A	512/8	1	A	64/8	0.56	A	512/128	0.56	A
**S121**	128/0.5	0.625	A	128/128	0.37	S	128/8	0.625	A	512/1	0.56	A	512/128	0.56	A
**S125**	64/1	0.625	A	128/256	0.5	S	64/4	0.37	S	128/4	0.75	A	256/128	0.625	A
**S127**	32/0.5	0.31	S	64/256	0.37	S	64/4	0.25	S	256/128	0.53	A	256/1	0.625	A
**S130**	512/0.5	0.75	A	512/64	0.56	A	128/16	0.625	A	256/16	0.75	A	512/512	0.625	A
**S131**	256/0.5	0.5	S	512/512	1	A	256/8	0.625	A	256/8	0.625	A	1024/512	0.75	A
**S135**	128/1	0.56	A	256/512	0.625	A	1024/4	0.75	A	256/8	0.625	A	1024/256	0.625	A
**S137**	128/1	0.56	A	1024/512	1	A	1024/2	0.75	A	512/4	0.75	A	512/512	0.5	S
**S141**	256/0.5	0.37	S	1024/512	1	A	256/8	0.625	A	256/8	0.625	A	1024/512	0.75	A
**S145**	128/0.5	0.625	A	128/256	0.625	A	512/8	1	A	64/8	0.56	A	512/128	0.56	A
**S160**	64/1	0.625	A	128/256	0.5	S	64/4	0.37	S	128/4	0.75	A	256/128	0.625	A
**S161**	256/0.5	0.37	S	1024/512	1	A	256/8	0.625	A	256/8	0.625	A	1024/512	0.75	A
**S162**	128/0.5	0.625	A	256/128	0.5	S	128/4	0.37	S	512/8	1	A	256/512	0.75	A
**S163**	256/0.5	0.37	S	1024/512	1	A	256/8	0.625	A	256/8	0.625	A	1024/512	0.75	A

S: synergism, A: additive effect, I: interpretation, FIC: fractional inhibitory concentration, AZM: Azithromycin, LZD: Linezolid, GEN: Gentamicin, AMI: Amikacin, CEF: Ceftriaxone, and CTX: Cefotaxime.
